# Effect of Cryo-Rolling on the Microstructural Evolution and Mechanical Properties of Ti-6Al-4V Alloy

**DOI:** 10.3390/ma18235296

**Published:** 2025-11-24

**Authors:** Vaibhav Gaur, Pargam Vashishtha, Biraj Kumar Sahoo, Ravi Kumar Bandari, Raj Das

**Affiliations:** 1 CSIR-National Metallurgical Laboratory, Jamshedpur 831007, India; biraj.nml@csir.res.in; 2 Academy of Scientific and Innovative Research (AcSIR), Ghaziabad 201002, India; pargamvashishtha@gmail.com; 3 School of Engineering, Royal Melbourne Institute of Technology (RMIT), Melbourne 3000, Australia; raj.das@rmit.edu.au

**Keywords:** Ti-6Al-4V, cryo-rolling, grain refinement, deformation mechanism, mechanical properties

## Abstract

Ti-6Al-4V is valued for its strength-to-weight ratio in engineering applications. Cryo-rolling at sub-zero temperatures enhances strength and hardness through grain refinement and dislocation build-up. The present study investigates the role of cryo-rolling on the microstructural characteristics and mechanical properties of the alloy, which undergoes various degrees of deformation followed by heating at 900 °C for selected samples. Microstructural analysis reveals grain elongation, sub-grain formation, deformation bands, and dislocation densification with increasing thickness reduction. Twinning dominates deformation at low strain, while dislocation slips take over at high strain because of the decrease in grain size, which makes the formation of new twins progressively more challenging. No metastable phase appears during cryo-rolling or heat treatment, as confirmed by X-ray diffraction. Cryo-rolled samples exhibit about 45% and 29% reduction in grain size and crystallite size, and 160% intensification in dislocation density. This leads to rises of 19%, 23%, and 10% in yield strength, tensile strength, and hardness, respectively, while ductility remains nearly constant across all cryo-rolled conditions. Cryo-rolling inhibits dynamic recovery and recrystallisation, so strengthening mainly results from grain refinement and dislocation accumulation. These findings suggest that cryo-rolling can improve the strength and hardness of Ti-6Al-4V, while maintaining ductility and providing new processing insights.

## 1. Introduction

Titanium and its alloys are known for their exceptional combination of mechanical and bio-mechanical properties, such as high specific strength, remarkable corrosion resistance, hardness, fatigue resistance, and high-temperature resistance [1,2,3], accompanied by notable biocompatibility [4,5,6]. These impressive qualities make them suitable for high-performance applications like aerospace components, the automotive sector, the chemical industries, and biomedical implants [7,8,9].

Ti-6Al-4V is a well-known alpha-beta titanium alloy [10]. Among all titanium alloys, it has become the most extensively used [11], accounting for approximately 50% of the total global production of titanium and its alloys [12]. It is primarily valued for its high strength, low density, corrosion resistance [13], and biocompatibility, making it suitable for biomedical applications [14,15,16] such as orthopedic [17] and dental implants [18,19]. Additionally, its low thermal conductivity and high melting point make it ideal for the aerospace sector [20,21], including critical structural components, jet engine parts [22], and landing gear elements [23]. The stability of the abovementioned properties and applications is due to its remarkable microstructure, which consists of an α + β dual phase [24].

In recent decades, improving mechanical properties has become a significant focus of metallurgical research. Microstructural characteristics, like grain size, dislocation density, and phase composition, play a crucial role in enhancing mechanical properties [25,26,27]. Over the past few years, studies have increasingly centred on innovative methods for producing ultrafine grains (UFG) to enhance these mechanical traits [28,29,30]. These UFG structures resulted in improved mechanical properties and corrosion resistance [31,32,33]. Several processing techniques have been incorporated into producing UFG in Ti-6Al-4V alloy. Sergueeva et al. [34] performed high-pressure torsion (HPT) to develop UFG in Ti-6Al-4V alloy to achieve high-strain-rate superplasticity. Zherebtsov et al. [35] demonstrated an increase in mechanical strength (25–40%) and fatigue resistance, along with a reduction in ductility, through the production of UFG in Ti-6Al-4V using the warm “abc” deformation method. Semenova et al. [36], Saitova et al. [37], Semenova et al. [38], Ko et al. [39] and Fernandes et al. [40] produced UFG through severe plastic deformation (SPD) with the help of equal channel angular pressing (ECAP), resulting in enhanced mechanical strength and fatigue properties, enhanced superplastic deformation behaviour and surface properties (for biomedical applications), respectively. Pazhanivel et al. [41] developed an ultra-fine bimodal structure using heat treatment of laser powder bed fusion (LPBF) produced Ti-6Al-4V. Li et al. [42] demonstrated the enhancement of corrosion resistance by creating a nanograined layer through rapid multiple rotation rolling on the surface of Ti-6Al-4V alloy. The grain size achieved through these processes ranges from 500 nm to 10 μm.

Yu et al. [43] demonstrated that the refinement of grain size correlates with the improvement of the second phase and an increase in dislocation density, thus contributing to superior mechanical properties. Gu et al. [44] examined the effects of cryogenic treatment. They clarified a reduction in the quantity of the β phase due to the transformation of metastable β into stable α and β phases. Furthermore, rolling at cryogenic temperatures has become a prominent technique for grain refinement, thereby enhancing the mechanical properties of titanium and its alloys [45].

Cryogenic treatment, particularly cryo-rolling, has recently drawn attention and emerged as a prominent technique for grain refinement. It refines the grains up to the level of UFG and nanograins. The rolling at sub-zero temperature helps in the suppression of dynamic recovery and recrystallization, which results in the rise in dislocation density and the formation of sub-grains, enhancement of mechanical properties through grain refinement and enhanced dislocation density via cryo-rolling of Ti-6Al-4V sheets. With the help of multiple cryogenic treatments, Kang et al. [46] demonstrated the reduction in the β phase in Ti-6Al-4V alloy, resulting in increased dislocation density and microstrain, which led to increased compressive strength, failure strain, and wear resistance of the alloy. Gu et al. [47] reported an increment in elongation accompanied by the strengthening of Ti-6Al-4V alloy, resulting from the reduction in precipitate particles after cryogenic treatment.

Despite extensive research conducted in recent years on Ti-6Al-4V for enhancing its properties, such as mechanical, chemical, thermal, corrosion resistance, and biocompatibility for applications like aerospace industries and biomedical implants, cryogenic treatment remains relatively uncommon, although a few studies have been undertaken to understand the influence of cryogenic rolling processes on the material’s mechanical properties and crystal structure behaviour; however, a significant research gap persists with respect to the evolution of microstructure and mechanical properties. This study aims to deepen the understanding of advanced material processing techniques and their potential impact on improving the performance of Ti-6Al-4V. The current investigation systematically explores the effects of cryogenic rolling at varying deformation percentages on the process-structure-property relationship of Ti-6Al-4V. A detailed microstructural analysis was conducted to identify phase morphology, deformation features, phase identification, crystallite size, and dislocation density. Furthermore, mechanical properties of the material were investigated through tensile tests and Vickers hardness measurements. A detailed correlation between microstructure and mechanical properties was established. Additionally, some preliminary work was conducted to examine the effect of post-heat treatment on cryo-rolled samples.

## 2. Materials and Methods

In the present study, a 10 mm thick Ti-6Al-4V (grade 5) plate, with the chemical composition outlined in Table 1, was utilized. Chemical analysis was conducted to accurately determine alloy composition and correlate it with the ASTM B 265–20a standard [48]. The elemental composition was determined using inductively coupled plasma-optical emission spectrometry (ICP-OES), while gas analysis was performed using Laboratory Equipment Corporation (LECO) techniques. Fine chips were produced using a drilling machine operating at low speed for ICP-OES analysis, whereas pins measuring 4 mm in diameter and 10 mm in length were fabricated using a manual lathe machine for LECO analysis.

**Figure 1:**
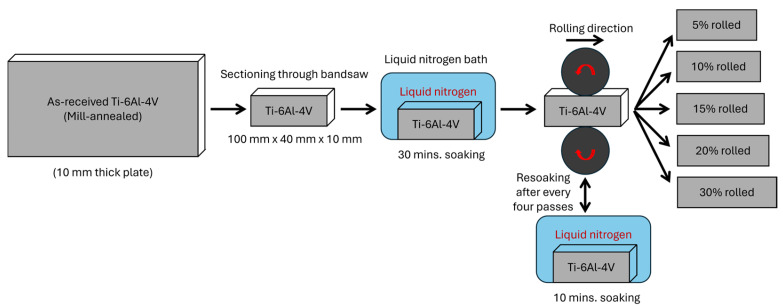
Schematic representation of the cryo-rolling process of Ti-6Al-4V alloy plate at different percentages of thickness reduction varying from 5% to 30%.

A total of six samples, each measuring 100 mm × 40 mm × 10 mm, were sectioned from the plate using a bandsaw, one for as-received investigation and five for cryo-rolling. Five samples were immersed in a liquid nitrogen bath at −196 °C for 30 min. Subsequently, specimens soaked in a liquid nitrogen bath were subjected to unidirectional multiple-pass rolling using AMECO’s laboratory-scale two-high rolling mill (Albert Mann Engineering Co. Ltd., Basildon, Essex, UK), which features a mechanically adjustable roll gap with a 220 mm roller diameter, with varying percentages of deformation ranging from 5% to 30% thickness reduction. The samples were re-soaked in liquid nitrogen for 10 min after every four passes to maintain processing at ambient temperature. The schematic of the cryo-rolling process is shown in Figure 1.

Specimens cryo-rolled at 15% and 30% were subjected to heat treatment in a Carbolite laboratory chamber furnace CWF 1300 (Hope, Derbyshire, UK) maintained at 900 °C for a soaking duration of 30 min, followed by air cooling. The purpose of heat treatment was to relieve stress and induce recrystallization.

Specimens in their cryo-rolled and heat-treated conditions were characterized using optical microscopy, scanning electron microscopy (SEM), energy dispersive X-ray spectroscopy (EDS), electron backscattered diffraction (EBSD), transmission electron microscopy (TEM) and X-ray diffraction (XRD). The plane selected for characterization across all conditions was bound by the rolling and transverse directions, commonly referred to as the rolling surface or plane. Optical microscopy of the surfaces of both as-received and cryo-rolled specimens, as well as post-heat-treated specimens, was performed using a Leica DM 2500 M optical microscope (Leica, Wetzlar, Germany). This analysis aimed to observe the microstructural changes resulting from cryo-rolling at different deformation percentages. SEM was conducted on the same conditioned samples using a Zeiss EVO 18 scanning electron microscope (Zeiss, Oberkochen, Germany). SEM aimed to observe microstructural variations in depth at higher magnifications resulting from cryo-rolling at various deformation percentages and post-heat-treatment. The elemental distribution resulting from the effects of cryo-rolling at different deformation percentages was analyzed using EDS on cryo-rolled specimens with a Zeiss EVO 18 scanning electron microscope.

EBSD and TEM of the selected samples were performed using a Hitachi Ultra-High-Resolution Schottky Scanning Electron Microscope (SU7000) (Hitachi, Tokyo, Japan), equipped with a field-emission gun (FEG), and a JEOL-2100F transmission electron microscope (JEOL, Tokyo, Japan), respectively, to identify the deformation mechanism. EBSD analysis was performed on TSL OIM software version 8.1. For TEM analysis, the samples were mechanically ground to a thickness of approximately 50 µm using SiC papers with grit sizes ranging from 120 to 2000, from which 3 mm discs were punched. These discs were subsequently thinned to electron transparency by perforation using a Struers Tenupol-5 twinjet electropolishing unit (Struers, Ballerup, Denmark). Electropolishing was carried out in an electrolyte containing 5 vol.% perchloric acid in methanol, cooled to −35 °C with liquid nitrogen, and operated at 25 V. The analysis was performed using the Gatan Digital Micrograph-3.4.

XRD analysis of as-received and cryo-rolled specimens was conducted using a Rigaku Ultima IV X-ray diffractometer (Rigaku, Tokyo, Japan) with a cobalt source (wavelength λ = 1.79 Å). Analysis was performed using PDXL-2.0 software to identify phases and ascertain their lattice parameters.

**Figure 2:**
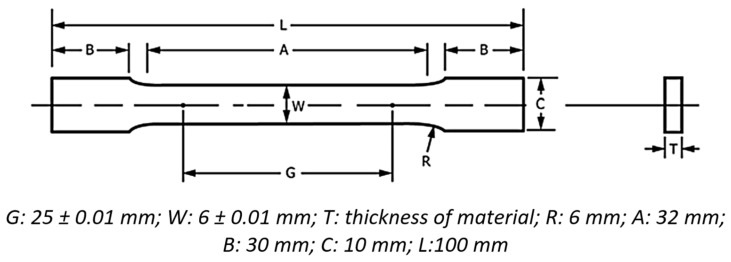
Flat tensile test specimen drawing (as per ASTM E8).

Mechanical testing included tensile and hardness tests. Uniaxial tensile test specimens were fabricated with the help of wire electrical discharge machining (EDM) in accordance with the ASTM E8 [49] international standard as shown in Figure 2. Subsequently, tensile testing was performed under each condition using an Instron Model 8501 universal testing machine (Instron, Norwood, MA, USA), operating at a constant strain rate of 0.2 mm/min. Vickers hardness tests were conducted on small specimens measuring 20 mm in length and 15 mm in width, with the longer dimensions aligned parallel to the rolling direction (RD). Hardness measurements were taken using a Vickers VH-50MD hardness-testing machine (Chennai Metco, Chennai, India), with indentations spaced 5 mm apart and a load of 30 Kgf applied for each indentation. These tests on the cryo-rolled specimens aimed to examine variations in mechanical properties, including strength, percentage elongation, and hardness, across different rolling percentages at sub-zero temperatures.

## 3. Results and Discussion

This section provides a systematic analysis of the impact of cryo-rolling on the microstructural evolution, deformation mechanisms, and mechanical properties of Ti-6Al-4V at deformation percentages ranging from 5% to 30%. The results are organized to establish a clear understanding of the structure–property relationship.

### 3.1. Microstructure Analysis

**Figure 3:**
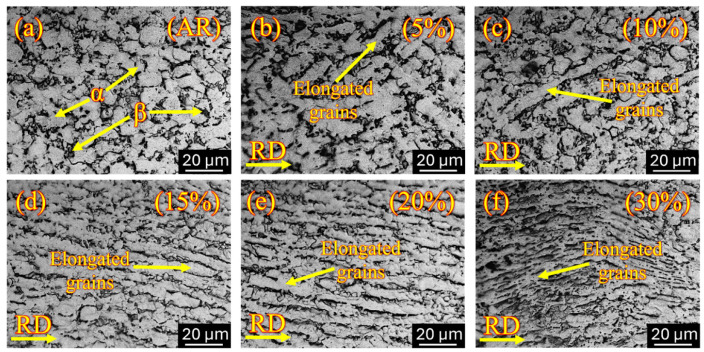
Optical micrography along the rolling direction (RD) of (**a**) as-received specimen shows equiaxed microstructure containing α grains in the matrix and β at the grain boundaries, and cryo-rolled specimens show grain elongations in (**b**) 5% deformation, (**c**) 10% deformation, (**d**) 15% deformation, (**e**) 20% deformation and (**f**) 30% deformation.

**Figure 4:**
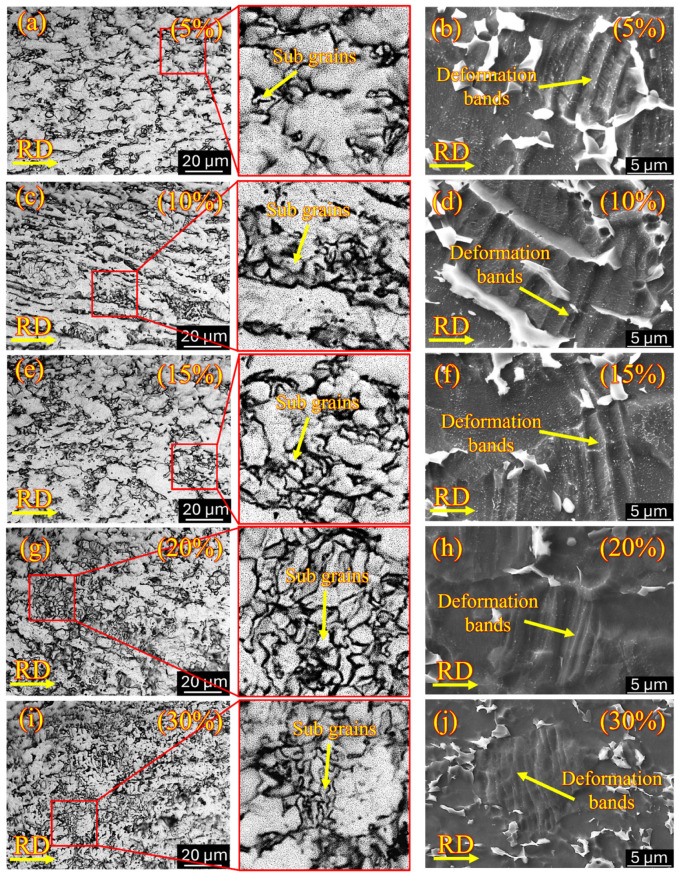
Optical micrography and SEM of cryo-rolled specimens: (**a**,**b**) 5% deformation, (**c**,**d**) 10% deformation, (**e**,**f**) 15% deformation, (**g**,**h**) 20% deformation and (**i**,**j**) 30% deformation showing formation of sub-grain structure and deformation bands, respectively.

The microstructural evolution, along with the internal grain structural characteristics of cryo-rolled Ti-6Al-4V specimens subjected to various degrees of plastic deformation, was systematically examined using optical microscopy and SEM, as illustrated in Figure 3 and Figure 4. As-received specimens exhibit a fine equiaxed structure of α-phase grains, characterized by an α + β dual-phase structure, as shown in Figure 3a, with the β phase predominantly distributed along the grain boundaries of the α phase. This suggests that the specimen is initially in a mill-annealed condition [50]. The measured grain size in as received condition is approximately 9.19 µm. In the optical micrographs and SEM images, α and β phases are distinguishable by their contrasting appearance. Bright and dark areas represent α and β phases, respectively, in optical microscopy, while the contrast is reversed in SEM images, where the β phase appears bright and the α phase appears dark. The initial microstructure of as-received specimen consists of prior α grains with an area fraction exceeding 95%, accompanied by a distribution of β phase at α grain boundaries, constituting a small fraction of less than 5%.

One of the key indicators of the cryo-rolling process is ongoing grain refinement, which is proportional to the deformation percentage. Upon cryo-rolling, substantial grain refinement is observed, with slightly elongated grains aligned along the rolling direction, accompanied by deformation bands as shown in Figure 3 and Figure 4. These microstructural features become increasingly prominent as the rolling percentage increases, indicating a progressive accumulation of plastic strain. The shearing mechanism plays a crucial role in the elongation of α grains during cryogenic treatment by forming shear bands [51]. At 5% cryo-rolling (Figure 3b), the initiation of grain elongation takes place within the microstructure, complemented by the appearance of lamellar deformation bands. However, there is not much change in overall grain morphology. By increasing the percentage of deformation from 10% (Figure 3c) to 15% (Figure 3d), further elongation of grains occurs almost parallel to the rolling direction due to the presence of localized shearing [52]. Further increasing the rolling percentage from 20% (Figure 3e) to 30% (Figure 3f) results in significantly elongated, well-defined grains, accompanied by densification of shear bands within the deformed structure. Due to the rolling in the cryogenic temperature regime, the recovery process is limited compared to that which occurs at ambient temperature [53].

Grain refinement takes place along with the formation of sub-grains, as shown in the highlighted zoomed section of Figure 4a,c,e,g,i. Apart from sub-grain formation, the formation of shear bands also plays a vital role in grain size reduction due to the rise in strain at high deformation percentages, which results from soft α and hard β phases present in the microstructure [54].

At deformation percentages from 5% (Figure 4a,b) to 10% (Figure 4c,d), the initiation of sub-grain formation takes place along with the development of localized deformation bands. Increasing the percentage of deformation from 15% (Figure 4e,f) to 20% (Figure 4g,h), the progressive development of sub-grain structure takes place, resulting in the densification of deformation bands within the grains with high intensity. This densification of deformation bands is caused by the accumulation of plastic strain during an increase in thickness reduction [55,56]. These bands endorse the storage of dislocations, promoting the localized shearing mechanism [57]. Finally, at 30% deformation (Figure 4i,j), the microstructure tries to achieve an ultrafine grain morphology, promoting the formation of a highly dense sub-grain structure along with an intricate network of deformation bands [58].

**Figure 5:**
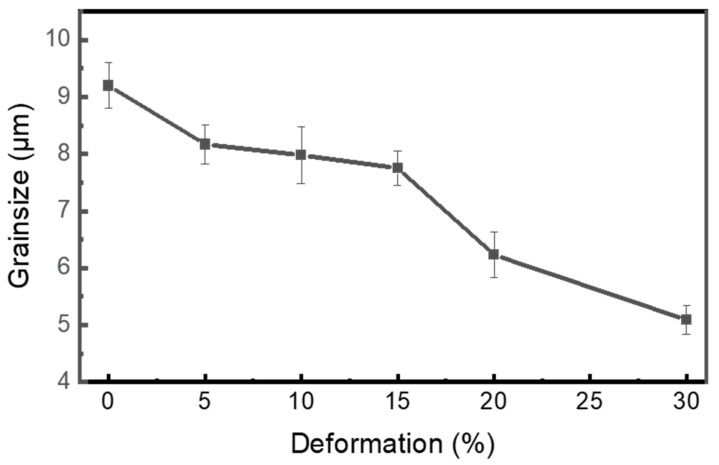
The graph represents the decreasing trend of grain size of as-received (AR), cryo-rolled and heat-treated (HT) specimens with respect to the increase in deformation percentages.

Figure 5 illustrates a quantitative analysis of grain size as a function of deformation percentage, showing a decreasing trend in grain size with increasing deformation. The grain size decreases from approximately 9 µm in the as-received condition to around 5 µm at the 30% cryo-rolling condition. This nearly 45% decrease in grain size indicates progressive grain refinement with increasing thickness reduction. A continuous decrease in grain size results from strain accumulation caused by lattice defects during deformation at cryogenic temperatures, in the presence of liquid nitrogen, where the suppression of dynamic recovery and recrystallization occurs. The continuous recrystallization, along with the subsequent formation of sub-grain structure and densification of deformation bands with increasing rolling percentage, represents the correlation between microstructure evolution and strain accumulation during the cryo-rolling process of Ti-6Al-4V, which is essential for enhancing mechanical properties.

**Figure 6:**
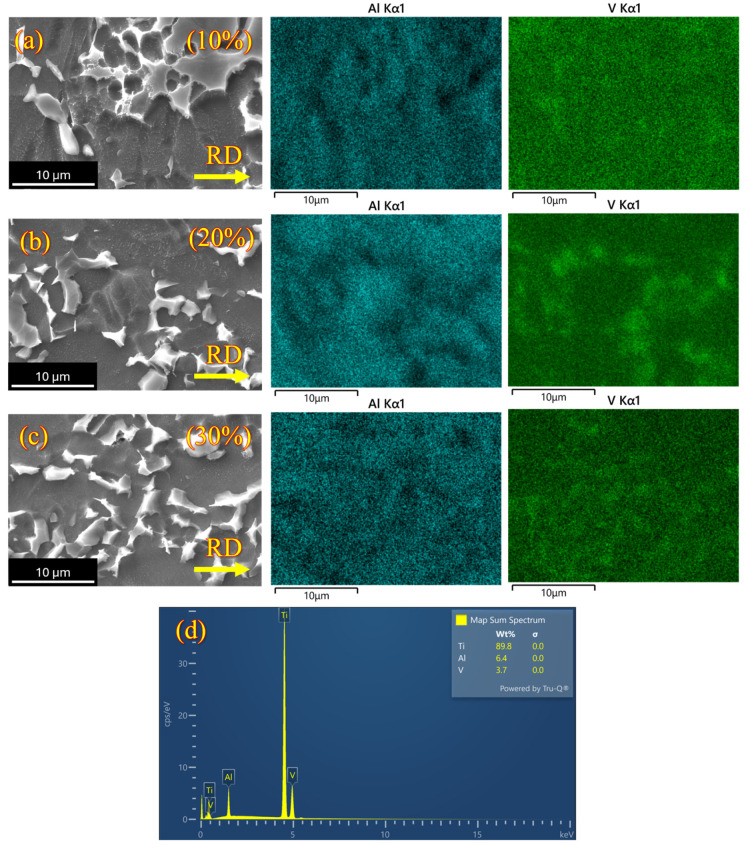
EDS map shows the fragmentation of β grains in cryo-rolled specimens in (**a**) 10% deformation, (**b**) 20% deformation, and (**c**) 30% deformation, along with (**d**) spectra of elements in cryo-rolled Ti-6Al-4V.

Elemental distribution across the specimen’s surface, along with the phase composition of aluminum and vanadium, was analyzed using EDS on samples that were cryo-rolled as shown in Figure 6a–c. Figure 6d represents a plot of elemental concentrations, indicating no significant changes in bulk elemental percentages resulting from cryo-rolling deformation. The retention of elemental consistency occurs because, during cryo-rolling, deformation is propagated through defect-induced mechanisms rather than diffusion-driven elemental redistribution. A notable increase in dislocation density, discussed in a later section, limits the elongation of β particles during deformation, leading to grain refinement and fragmentation of the β phase particles under cryo-rolled conditions.

### 3.2. Deformation Mechanism

To identify the deformation mechanism during the cryo-rolling of Ti-6Al-4V, EBSD and TEM analyses were performed on samples with three different deformation percentages. The selection of the samples is based on low (10%), medium (20%), and high (30%) thickness reductions. The strain accumulation increases with the rolling percentage due to the cryogenic conditions. Rolling at such low temperature limits strain relief, as no heat is generated during deformation. Consequently, this restricts dislocation movement, leading to dislocation accumulation, grain refinement, fragmentation of β grains, and the formation of sub-grains. In Ti-6Al-4V, both twinning and dislocation slip contribute to deformation at cryogenic temperatures. Initial deformation at lower strains occurs primarily through twinning, which is dominant due to the limited number of slip systems. In comparison, dislocation slip becomes more significant at higher strains with the reduction in grain size. During the cryo-rolling process, grain refinement is significantly achieved by the deformation twinning. At the same time, grain size plays an essential role in the tendency for the formation of twinning, with an inverse relationship [59,60,61]. The deformation mechanism shifts from twinning to dislocation activity with the increase in deformation percentage [62,63] due to the refinement of grains and the continuous generation and multiplication of dislocations. This causes an increase in dislocation density, which inhibits twin propagation, and the formation of new twins becomes more complex, leading to a reduction in twin fraction.

**Figure 7:**
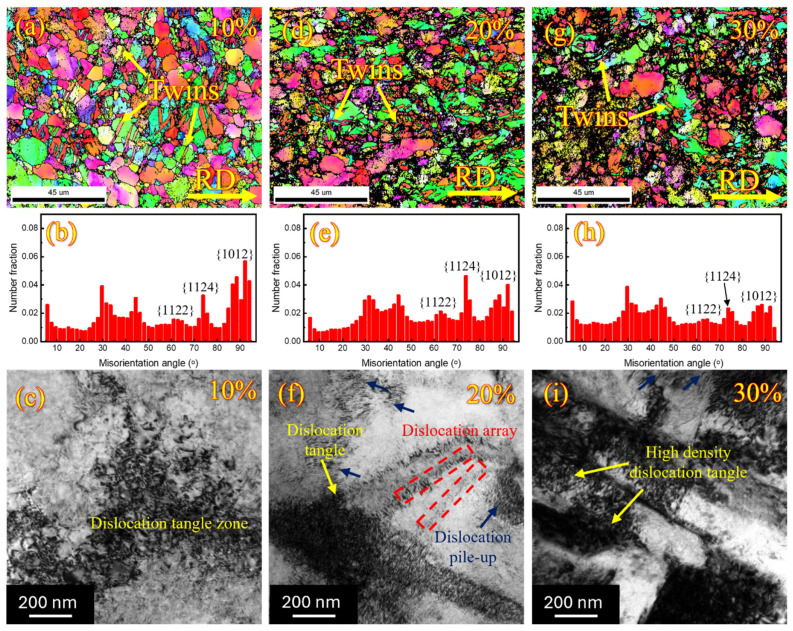
Inverse pole figure (IPF) maps, misorientation angle distribution graphs, and bright-field TEM micrographs of cryo-rolled Ti-6Al-4V samples deformed by (**a**–**c**) 10%, (**d**–**f**) 20%, and (**g**–**i**) 30%, respectively. The IPF maps reveal the presence of deformation twins, which become less prominent with increasing deformation. Bright-field TEM micrographs show the densification of dislocation features with the same.

For analysis of the activated twinning system during cryo-rolling, the misorientation angle and the rotation axis related to each twinning system are derived from the EBSD results, as shown in Table 2. They are consistent with findings reported in previous studies [64,65,66,67]. The EBSD and TEM analyses of the samples were performed along the rolling surface and are shown in Figure 7. EBSD data include inverse pole figure (IPF) maps and misorientation angle distribution graphs, which display twins and their corresponding misorientation angles. Meanwhile, bright-field TEM micrographs reveal dislocation features. The presence of both tensile and compressive primary twins, along with secondary twins, has been observed in the IPF and verified through misorientation distribution graphs, accompanied by a coincident-site lattice (CSL) boundary ∑13a (~30°). When identical or different types of twins occur in one or multiple variants in the grain, they are termed primary twins. On the other hand, when a twin nucleates inside another pre-existing twin, they are known as secondary or double twins. The peak around 30º along the c-axis with the rotation axis [0001] has also appeared in several previous deformation studies of hexagonal close-packed (HCP) metals over the past several years [68,69,70,71]. The observed interface for this peak is not characterized as a twin boundary or a boundary separating twin variants [72], so the cause behind its occurrence remains uncertain [70,73].

The presence of deformation twins has been significantly observed at a low strain of 10% deformation (Figure 7a), indicating that at this low strain, deformation occurs predominantly through twinning. The fraction of tensile twins {10$\bar{1}$2} <10$\bar{1}\bar{1}$> occurs at the misorienting angle of around 86.7°, which is higher than that of compressive twins. This shows that at low deformation percentages, deformation takes place through the combination of both tensile and compressive twins, with tensile twins dominating the compressive twins, as shown in Figure 7b. It indicates that the proportion of grains suitable for compressive twinning is lower than for tensile twinning. Also, the presence of secondary twins takes place at 44.4°. These twins result from the nucleation of {10$\bar{1}$2} <10$\bar{1}\bar{1}$> type tensile twins within the {11$\bar{2}$2} <11$\bar{2}\bar{3}$> type compressive twins. These twins correspond to the rotation axis <1$\bar{5}$43> [65]. Additionally, the bright-field TEM micrograph shows a low-density dislocation zone (Figure 7c). When the thickness reduction increases to 20%, grain fragmentation occurs, which restricts the formation of new twins and leads to twinning saturation [74]. This saturation effect is visible as a reduction in the fraction of {10$\bar{1}$2} <10$\bar{1}\bar{1}$> tensile twins taking place, while the fraction of compressive twins increases slightly, as shown in Figure 7d,e. This indicates that with the increase in deformation percentage, compressive twins become more favourable. At the same time, the non-indexed points shown in black in Figure 7d also increase, representing the increase in structural defects. As the twinning fraction decreases, densification of dislocation features such as dislocation pile up, dislocation array and dislocation tangle take place, as shown in Figure 7f. Further increase in rolling percentage to 30% enhances heterogeneity within the microstructure due to the presence of some coarse grains; further growth of non-indexed points signifies higher structural defect density, as shown in Figure 7g. Along with a reduction in the fraction of both compressive as well as tensile twins (Figure 7h), formation of high-density dislocations takes place within the material (Figure 7i). Deformation mechanism transformed from twinning-induced mechanism to dislocation slip mechanism happens as the twinning becomes less energetically favourable with an increasing strain [75].

### 3.3. Phase Analysis After Cryo-Rolling

**Figure 8:**
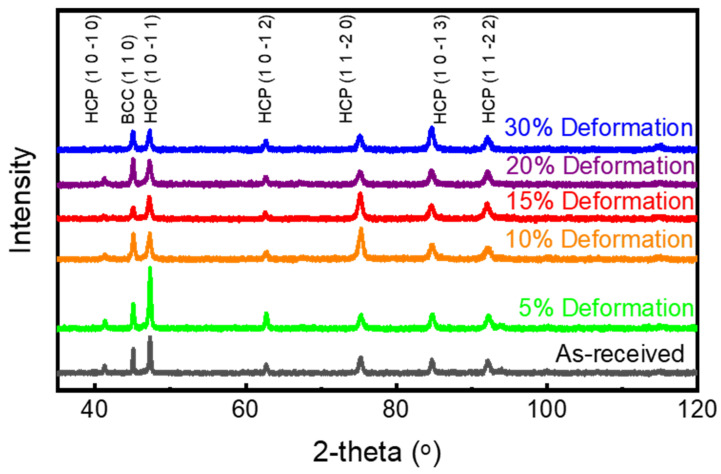
XRD plot of as-received and cryo-rolled specimens represents the α and β phases in Ti-6Al-4V.

The study of crystallographic evolution, lattice parameter changes, crystallite size, and dislocation density in Ti-6Al-4V subjected to cryo-rolling at different deformation percentages was performed using X-ray diffraction (XRD) analysis. The diffractograms for cryo-rolled samples are presented in Figure 8. All peaks in these XRD patterns are exclusively indexed to the hexagonal close-packed (HCP) α and body-centred cubic (BCC) β phases of titanium, confirming the persistence of the alloy’s dual-phase structure, showing the absence of any phase transformation induced in either condition. However, minute peak broadening and variations in relative peak intensity indicate that microstructural refinement and lattice strain accumulation have occurred due to cryo-rolling. This peak broadening mainly results from the combined effects of two interconnected mechanisms.

- A reduction in crystallite size, caused by the formation of dislocation cells, and
- An increase in micro-strain, resulting from the increase in dislocation density, occurs during cryo-rolling processing.

These XRD results indicate an interlink between the piling up of dislocations and grain refinement resulting from cryo-rolling deformation, providing support for the enhancement in mechanical properties.

With the help of XRD diffractograms, the lattice parameters of both α and β phases of Ti-6Al-4V have been calculated using Bragg’s law (Equation (1)) along with the crystallographic relationships between lattice parameters and interplanar spacing for HCP (Equation (2)) and cubic (Equation (3)) systems. The HCP α-phase exhibits a c/a ratio of about 1.59, while the BCC β-phase features a lattice constant of approximately a = 3.32 Å. Throughout the cryo-rolling process, no significant changes are observed, indicating that the chemical composition and phase stability of the alloy remain stable during processing. This consistency in the lattice parameter indicates the absence of solid solution redistribution and elemental partitioning during processing, suggesting that the microstructural evolution and enhancement in mechanical properties are driven by defect-induced mechanisms rather than changes in elemental concentration.

(1) $$2d_{hkl}sin\theta =\lambda \;\;\;\;\;\;\;\;(\mathrm{Bragg}’s\;\mathrm{Law})$$

(2) $$\frac{1}{d_{hkl}^{2}}=\frac{4}{3}(\frac{h^{2}+hk+k^{2}}{a^{2}})+\frac{l^{2}}{c^{2}}\;\;(\mathrm{for}\;\mathrm{HPC})$$

(3) $$\frac{1}{d_{hkl}^{2}}=\frac{h^{2}+k^{2}+l^{2}}{a^{2}}\;\;\;(\mathrm{for}\;\mathrm{Cubic})$$

where d_hkl_ is the interplanar spacing (Å), 2θ is the Bragg’s diffraction angle (in degrees), λ is the wavelength used, and (a, b, c) and (h, k, l) are the lattice parameters and Miller indices, respectively.

#### 3.3.1. Crystallite Size

Crystallite size was calculated with the help of Williamson–Hall (W–H) method (Equation (4)), which deconvolutes peak broadening into contributions from crystallite size and microstrain using the following equation [76,77]:

(4) $$\beta \;cos\theta =\frac{k\lambda }{D}+4\;\varepsilon \;sin\theta$$

where β is the full width at half maximum (FWHM) in radians, θ is the Bragg angle, k is the shape factor (typically 0.9), λ is the X-ray wavelength (1.79 Å for Co-Kα), D is the crystallite size, and ε is the lattice strain.

**Figure 9:**
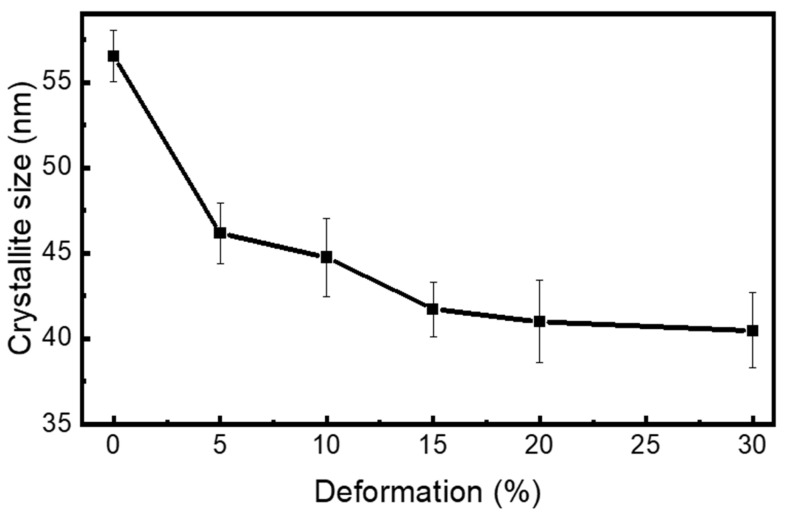
The graph represents the decreasing trend of crystallite size of as-received (AR) and cryo-rolled specimens with respect to the increase in deformation percentages.

A reduction of around 29% in crystallite size is observed as the deformation percentage increases, as shown in Figure 9. The average crystallite size is about 57 nm in the as-received condition and gradually decreases with increasing rolling percentage, reaching approximately 40 nm at 30% cryo-rolling. This reduction results from the suppression of dynamic recovery at cryogenic temperatures, which leads to the annihilation of dislocation rearrangements [78]. Consequently, dislocations accumulate within grains, forming smaller coherent domains that decrease the crystallite size.

#### 3.3.2. Dislocation Density

Dislocation density (ρ) was estimated for the total dislocations in a crystalline material by examining diffraction peak broadening. This approach relies on analyzing the line broadening of XRD peaks, caused by reductions in crystallite size and lattice strain that occur during plastic deformation. The dislocation density (ρ) is subsequently calculated using the lattice strain (ε) and crystallite size (D) through the following equation [79,80].

(5) $$\rho =\frac{2\surd 3\;\varepsilon }{bD}$$

where ρ is the dislocation density (m^−2^), ε is the lattice strain (dimensionless), b is the Burgers vector (m), and D is the crystallite size (m).

**Figure 10:**
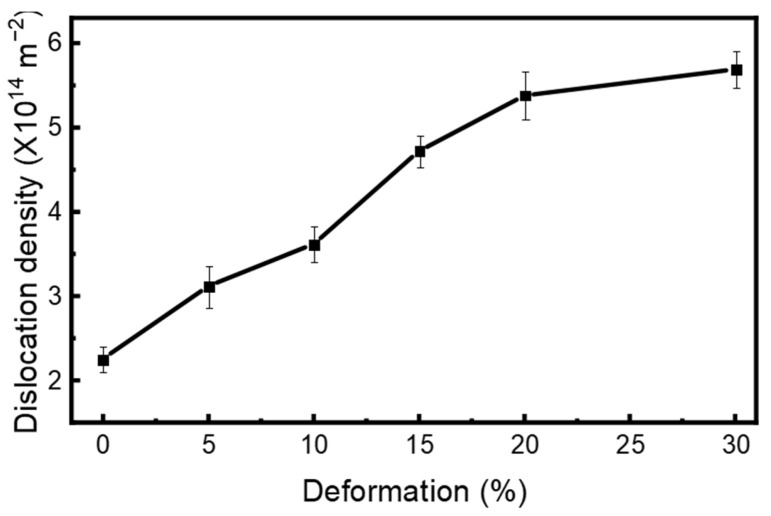
The graph represents the increasing trend of dislocation density of as-received (AR) and cryo-rolled specimens with respect to the increase in deformation percentages.

With increasing deformation percentage, a trend of increasing dislocation density is observed, as shown in Figure 10. Dislocation density approximately increases from 2.26 × 10^14^ m^−2^ in the as-received condition to 5.69 × 10^14^ m^−2^ at the 30% cryo-rolling condition, representing nearly 160% enhancement in dislocation density. During plastic deformation at −196 °C, dislocation climb or cross-slip is restricted by insufficient thermal energy, thereby inhibiting dynamic recovery. Consequently, dislocations are continuously generated and accumulated rather than being annihilated. This combined effect of reduced dislocation mobility and piling up of dislocations at cryogenic temperatures leads to a progressive increase in dislocation density [81]. The observed increase in dislocation density directly contributes to grain refinement and strength enhancement [82], which is also consistent with the Taylor hardening mechanism [83].

### 3.4. Mechanical Properties Measurement

**Figure 11:**
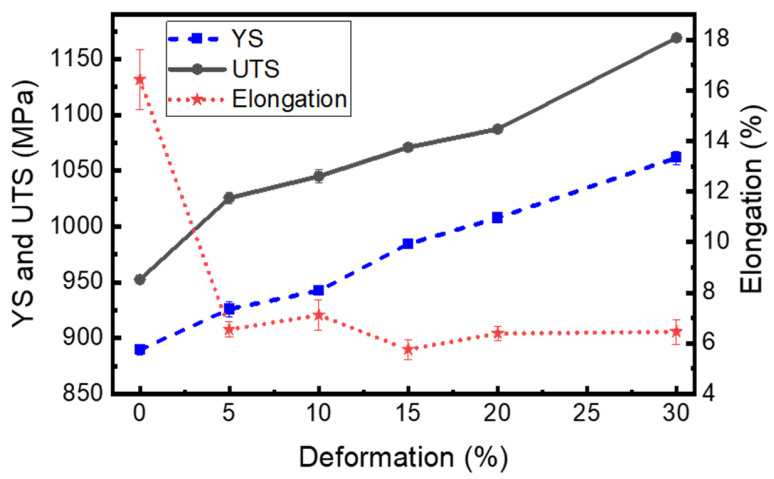
The graph represents the engineering stress–strain graph for as-received (AR) and cryo-rolled specimens at different percentages of deformation from 5% to 30% corresponding to an increasing trend of yield strength (YS) and ultimate tensile strength (UTS), with a drop in elongation trend.

**Figure 12:**
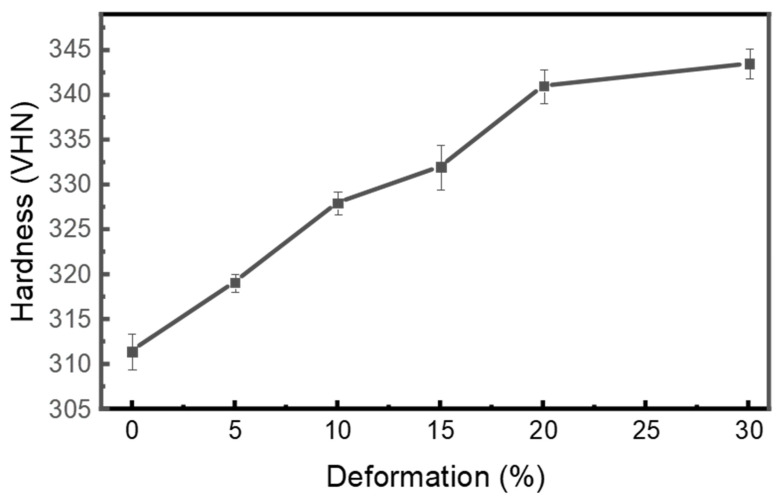
The graph represents the increasing trend of hardness of as-received (AR), cryo-rolled and heat-treated (HT) specimens with respect to the increase in deformation percentages.

To examine the mechanical properties and elongation behaviour of Ti-6Al-4V alloy having undergone different levels of cryo-rolling, uniaxial tensile and Vickers hardness tests were conducted on both as-received and cryo-rolled specimens at room temperature. This investigation aimed to evaluate the influence of cryo-rolling on the material’s strength, ductility, and hardness. The outcomes of these tests are presented in Figure 11 and Figure 12 and summarized in Table 3 and Table 4, respectively.

#### 3.4.1. Tensile Properties

During the rolling process, the relationship between strength and ductility remains one of the most critical material aspects. Cryo-rolling is a well-known method used to achieve ultrafine grains, resulting in high strength at the expense of ductility, as the plastic deformation process is governed by dislocation slip [84]. Figure 11 quantitatively illustrates the relationship between yield strength (YS), ultimate tensile strength (UTS), and percentage elongation for Ti-6Al-4V under various cryo-rolling deformation percentages, with corresponding numerical values listed in Table 3. The as-received sample exhibits a YS of 889.63 MPa, a UTS of 952.65 MPa, and 16.43% ductility, confirming its mill-annealed condition for Ti-6Al-4V.

An increase in deformation percentage leads to significant strain hardening, as shown in Figure 11, resulting in a corresponding increase in strength. In contrast, there is no appreciable change in the elongation percentage for cryo-rolled specimens. With rolling progress, a reduction in thickness occurs, accompanied by an increase in dislocation density, along with grain refinement and a decrease in crystallite size, resulting in the gradual increase in YS and UTS with decreasing thickness, reaching a maximum YS of 1061.66 MPa and a maximum UTS of 1169.10 MPa at 30% cryo-rolled deformation, indicating around 19% and 23% increase in YS and UTS, respectively. Although there is a sudden drop in percentage elongation under cryo-rolling conditions, the effect on elongation percentage of cryo-rolled specimens is less consistent, as it shows a wavy nature, but remains more or less constant. Du et al. [85] and Xu et al. [86] also observed a similar trend of ductility in their studies, with a constant increase in strength. This strength growth results from multiple factors contributing to deformation-induced strengthening mechanisms [87,88,89].

- Twinning induced strengthening occurs at the early stages of deformation, followed by dislocation strengthening as dislocation density increases with an increasing percentage of cryogenic deformation.
- Grain boundary strengthening occurs through the Hall–Petch effect, which results from microstructural refinement and elongation of α grains, and
- Low processing temperature during cryo-rolling hinders dynamic recovery by causing high strain energy retention and stabilizing deformation structures.

Although twinning aids in refining grains and increasing strength, grain fragmentation causes twins to saturate in the early stages of deformation. As dislocation density rises, it suppresses the growth of twins, making it progressively more challenging to form new ones [90]. During the initial stage of thickness reduction, the development of high-intensity twins contributes to the material’s brittleness, resulting in a sudden decrease in percentage elongation. The percentage elongation reduced from 16.43% for the as-received specimen to nearly 6.5% under cryo-rolling conditions, with a thickness reduction from 5% to 30%. This indicates restricted dislocation mobility, grain refinement, and work hardening resulting from strain-induced deformation and the accumulation of internal stresses, which suppress further plastic deformation [91,92]. This effect is intensified by shear localization and deformation banding, as observed in SEM analysis, where concentrated stress into a narrow region promotes early failure and decreases uniform plastic deformation.

#### 3.4.2. Hardness Behaviour

As shown in Figure 12 and Table 4, the Vickers hardness results exhibit a consistent increase in hardness with higher rolling percentages, from 311.5 Vickers hardness number (VHN) in the as-received condition to 343.5 VHN after 30% cryo-rolled deformation, indicating nearly a 10% increase in hardness. This increasing trend in hardness aligns with the strength trend observed in the tensile test, resulting from dislocation pileup and grain refinement. It confirms that the enhancement of both localized and bulk plastic deformation takes place due to cryo-rolling [93]. Fragmentation of the β-phase also plays a crucial role in the increasing trend of hardness with respect to the rise in deformation percentage [94], as the finer β-phase promotes the pinning effect of dislocations, resulting in the tangling of dislocations, which restricts further deformation [95]. The maximum hardness at 30% deformation is attributed to a fine crystallite size and high dislocation density, as indicated by XRD analysis.

### 3.5. Effect of Post-Heat Treatment on Cryo-Rolling

**Figure 13:**
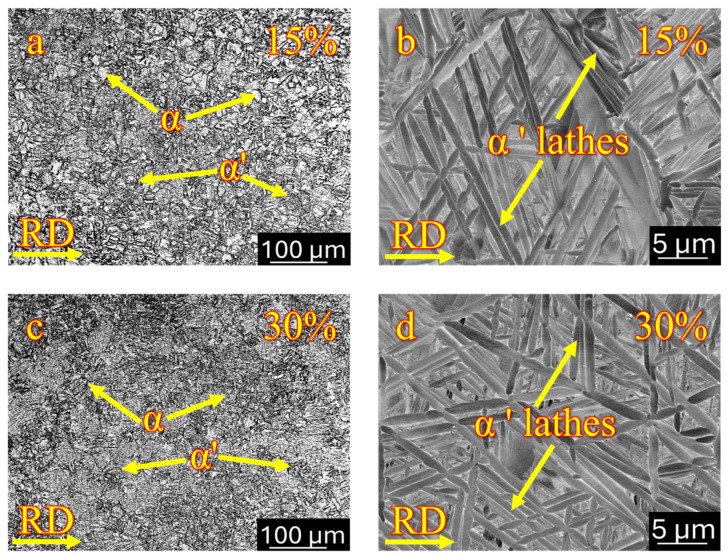
Optical micrography and SEM show duplex microstructure containing primary α (α) and secondary α (α’) in lathe morphology in cryo-rolled specimens resulting from post-heat treatment (**a**,**b**) 15% deformation and (**c**,**d**) 30% deformation.

Two specimens were selected for the post-heat-treatment process based on the medium (15%) and the highest (30%) deformation percentages. Cryo-rolled samples, followed by heat treatments at 15% and 30% thickness reduction, displayed a duplex microstructure as shown in Figure 13a (15% cryo-rolled followed by heat treatment) and 13c (30% cryo-rolled followed by heat treatment). The formation of a duplex microstructure took place due to partial recovery and recrystallisation resulting from post-deformation thermal exposure. A slight difference was observed between heat-treated and as-received samples, with heat-treated samples exhibiting marginally coarser grains than non-heat-treated ones. The average grain size was approximately 8 µm and 7 µm at the equivalent deformation percentages of 15% and 30% post-heat-treated conditions, respectively. Along with the grain coarsening, partial recovery and recrystallisation aided in eliminating deformation bands and rolling-induced grain elongation. This occurred due to the initiation of static recrystallization and grain growth during heat treatment. The elimination of the fragmented structure occurred through the nucleation of strain-free grains. A significant reduction in internal stresses and defects occurred during the soaking period at elevated temperatures, due to the stabilization of microstructures caused by partial recovery and recrystallization. The evolution of the final microstructure is strongly influenced by the cooling rate employed during the heat treatment, which plays a crucial role in the phase transformation mechanism. Cooling at room temperature provided sufficient time for the secondary α (α’) to nucleate and grow within the β matrix, resulting in the formation of a bimodal structure [96]. Nucleation of the secondary α began preferentially at the grain boundary, with growth progressing into the β matrix in the form of a lamellar structure, as shown in Figure 13b (15% cryo-rolled followed by heat treatment) and 13d (30% cryo-rolled followed by heat treatment).

**Figure 14:**
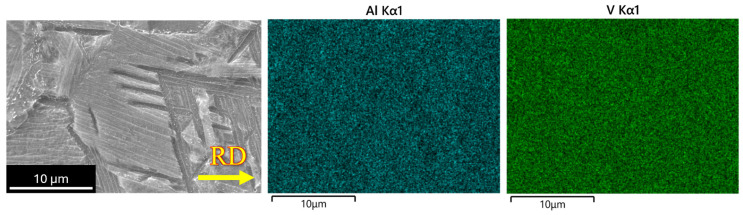
EDS map of the post-heat-treated specimen shows the absence of phase segregation.

**Figure 15:**
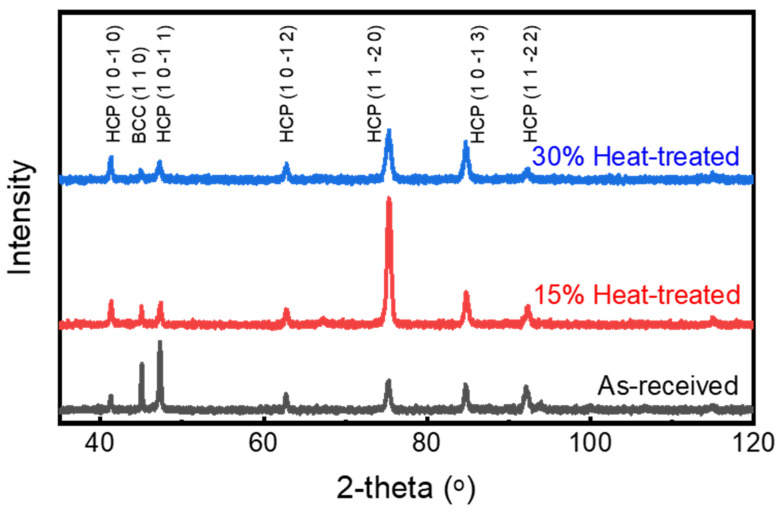
XRD plot of as-received and post-heat-treated (HT) specimens represents the α and β phases in Ti-6Al-4V.

Elemental distribution across the specimen’s surface for the post-heat-treated condition exhibits no signature of any phase segregation, as shown in Figure 14, attributed to recrystallization. Deformation-induced microstructural heterogeneity is effectively counteracted by the recrystallization process, resulting in homogeneity in microstructure by ensuring a uniform distribution of both α and β phases throughout the microstructure, a finding confirmed by optical and SEM analysis. Along with the surface and subsurface characteristics, bulk analysis was performed using XRD for phase identification during post-heat-treatment processing. The diffractograms for 15% and 30% post-heat-treated samples are shown in Figure 15, along with the as-received condition. These results are similar to those obtained under cryo-rolled conditions, indicating no change in the chemical composition or phase stability. This demonstrates that the processing did not induce elemental partitioning or the redistribution of solid solutions.

In post-heat-treated conditions, hardness decreases for both 15% (317.5 VHN) and 30% deformation (319.5 VHN) with respect to their respective non-heat-treated conditions. This reduction is attributed to the recovery and recrystallization process, which promotes dislocation rearrangement and annihilation, thereby releasing stored strain energy and contributing to material softening by lowering lattice strain. Although the hardness decreases, it remains higher than in the as-received condition (311.5 VHN), indicating that partial retention of strain-hardened microstructures persists within the material.

## 4. Conclusions

This study investigated the impact of cryogenic rolling on the microstructure evolution of Ti-6al-4v, including its deformation mechanism and mechanical properties. These analyses resulted in the following conclusions:

- Rolling at liquid nitrogen temperature suppressed dynamic recovery, enhancing dislocation density and subsequently grain refinement took place. The results obtained from the optical microscopy, SEM and EDS analysis show an intensification in elongated grains, reduction in grain size, sub-grain formation, densification of deformation bands and fragmentation of β grains with the increase in rolling percentage.
- Due to the limitations of slip systems, at lower strain, initiation of deformation took place via twinning. As the strain increases with an increase in thickness reduction, twinning became less favourable, and the formation of new twins became increasingly difficult. Twinning-induced deformation transformed into deformation via dislocation slip, with the formation of high-density dislocations.
- The presence of α (HCP) and β (BCC) phases has been verified through XRD. There is no phase transformation or formation of any metastable phases that take place during cryo-rolling. With increasing strain, a slight peak broadening and intensity changes have been identified due to the microstructural refinement. Williamson–Hall analysis is used to quantify the reduction in crystallite size and enhancement in dislocation density due to strain hardening with increasing rolling percentage.
- Enhancement of the mechanical properties like YS, UTS and hardness has been observed via tensile and hardness tests, respectively, with increasing deformation percentage, while the ductility corresponding to all the cryo-rolled samples remains relatively stable, resulting from the simultaneous occurrence of limited dislocation movement and higher internal strain energy and fragmentation of β phase.
- During heat treatment following cryo-rolling, partial recovery and recrystallization facilitated the formation of a duplex microstructure and the removal of internal stains, leading to grain coarsening, homogeneous distribution of α and β phases throughout the microstructure and a reduction in the material’s hardness.
- The relationship between microstructure evolution, deformation mechanisms, and mechanical properties demonstrates the effect of cryo-rolling on modifying grain boundaries and dislocations in Ti-6Al-4V. These findings resemble a strong bond between the structure, property and performance, signifying the role of deformation mechanisms in the enhancement of mechanical properties during cryo-rolling.

## Figures and Tables

**Table 1:** Chemical composition of as-received Ti-6Al-4V plate.

Elements	Al (wt.%)	V (wt.%)	Fe (wt.%)	C (wt.%)	N (ppm)	O (ppm)	H (ppm)	Ti (wt.%)
**Composition**	6.2	3.8	0.08	0.05	4	50	62.7	Balance

**Table 2:** Deformation twinning system.

Twinning Type	Twinning System	Rotation Axis	Misorientation Angle (°)
Tensile	{$10\bar{1}$2} <10$\bar{1}\bar{1}$>	<$1\bar{5}43$>	44.4
Compressive	{$11\bar{2}$2} <$11\bar{2}\bar{3}$>	<$10\bar{1}$0>	64.6
Compressive	{$11\bar{2}$4} <$22\bar{4}\bar{3}$>	<$10\bar{1}$0>	74.5
Tensile	{$10\bar{1}$2} <$10\bar{1}\bar{1}$>	<$11\bar{2}$0>	86.7

**Table 3:** Tensile properties of as-received (AR) and cryo-rolled specimens at different percentages of deformation from 5% to 30%.

Specimen	AR	5%	10%	15%	20%	30%
**YS** **(MPa)**	889.63 ± 5	925.91 ± 7	942.56 ± 3	984.34 ± 3	1007.93 ± 4	1061.66 ± 6
**UTS** **(MPa)**	952.65 ± 2	1025.59 ± 5	1045.07 ± 6	1071.09 ± 3	1087.42 ± 4	1169.10 ± 4
**Elongation** **(%)**	16.43 ± 1.2	6.55 ± 0.3	7.12 ± 0.6	5.76 ± 0.4	6.39 ± 0.3	6.45 ± 0.5

**Table 4:** Hardness of as-received (AR), cryo-rolled and heat-treated (HT) specimens.

Hardness	AR	5%	10%	15%	20%	30%
**VHN**	311.5 ± 2	319.17 ± 1	328 ± 1.3	332 ± 2.5	341 ± 1.9	343.5 ± 1.7

## Data Availability

The original contributions presented in this study are included in the article. Further inquiries can be directed to the corresponding authors.

## Abbreviations

The following abbreviations are used in this manuscript:

UFG	Ultrafine grains
HPT	High-pressure torsion
SPD	Severe plastic deformation
ECAP	Equal channel angular pressing
LPBF	Laser powder bed fusion
ICP-OES	Inductively coupled plasma-optical emission spectrometry
LECO	Laboratory Equipment Corporation
SEM	Scanning electron microscopy
EDS	Energy dispersive X-ray spectroscopy
EBSD	Electron backscattered diffraction
TEM	Transmission electron microscopy
XRD	X-ray diffraction
FEG	Field-emission gun
EDM	Electrical discharge machining
RD	Rolling direction
PPM	Parts per million
AR	As received
HT	Heat-treated
IPF	Inverse pole figure
CSL	Coincident-site lattice
HCP	Hexagonal close-packed
BCC	Body-centred cubic
WH	Williamson hall
FWHM	Full width at half maximum
YS	Yield strength
UTS	Ultimate tensile strength
VHN	Vickers hardness number
